# Prediction of early recovery of graft function after living donor liver transplantation in children

**DOI:** 10.1038/s41598-024-60211-6

**Published:** 2024-04-24

**Authors:** Bingqian Tan, Chenyu Yang, Jiqiang Hu, Huiwu Xing, Mingman Zhang

**Affiliations:** 1 Department of Hepatobiliary Surgery Children’s Hospital of Chongqing Medical University, Chongqing Key Laboratory of Pediatrics, National Clinical Research Center for Child Health and Disorders, Ministry of Education Key Laboratory of Child Development and Disorders, Chongqing, 400000 China; 2 Department of Pediatric Surgery, The First Affiliated Hospital of Zhengzhou University, Zhengzhou, 450000 Henan China

**Keywords:** Living donor liver transplantation, Children, Graft function recovery, Nomogram, Ambispective cohort study, Paediatric research, Computational models, Liver, Liver diseases, Risk factors

## Abstract

For end-stage liver disease in children, living donor liver transplantation (LDLT) is often the important standard curative treatment. However, there is a lack of research on early recovery of graft function after pediatric LDLT. This is a single-center, ambispective cohort study. We collected the demographic and clinicopathological data of donors and recipients, and determined the risk factors of postoperative delayed recovery of hepatic function (DRHF) by univariate and multivariate *Logistic* analyses. 181 cases were included in the retrospective cohort and 50 cases in the prospective cohort. The incidence of DRHF after LDLT in children was 29.4%, and DRHF could well evaluate the early recovery of graft function after LDLT. Through *Logistic* analyses and AIC score, preoperative liver function of donors, ischemia duration level of the liver graft, *Ln* (Cr of recipients before operation) and *Ln* (TB of recipients on the 3rd day after operation) were predictive indicators for DRHF after LDLT in children. Using the above factors, we constructed a predictive model to evaluate the incidence of postoperative DRHF. Self-verification and prospective internal verification showed that this prediction model had good accuracy and clinical applicability. In conclusion, we pointed many risk factors for early delayed recovery of graft function after LDLT in children, and developed a visual and personalized predictive model for them, offering valuable insights for clinical management.

## Introduction

Although great progress has been made in hepatitis B vaccines, antiviral drugs and surgery, the global burden of liver disease is still increasing, and the morbidity and mortality of liver disease are increasing year by year, especially in less developed regions^1–5^. According to incomplete statistics, 120 million people worldwide suffer from end-stage liver disease, resulting in more than 2 million deaths each year^1,2^. Not only that, liver disease increases the risk of cardiovascular disease, and has substantial effects on pre-adolescent health^6,7^.

For end-stage liver disease, palliative treatment is not ideal and lacks of evidence-based medical evidence, so liver transplantation (LT) is often the only standard curative treatment^8,9^. However, the severe shortage of donors has greatly tethered the development of LT and remains a great challenge worldwide. Living donor LT (LDLT), split LT, domino LT, expanded criteria donor LT and other operations have expanded the source of liver grafts and decreased waiting list mortality^10^. Fortunately, children with end-stage liver disease can benefit from grafts donated by their parents, and LDLT has become one of the most important treatment options for them, especially in Asia^11^.

With the development of LDLT, long-term prognoses and graft survival rates have been satisfactory in children after operation^10,12,13^. In the perioperative period, the recovery of patients is closely related to the length of hospital stay, hospitalization cost and long-term prognosis, especially organ transplantation, cancer-related surgery^14–16^. Postoperative graft function recovery directly reflects the graft survival rate and is closely related to the long-term quality of life, which is one of the keys to the success of organ transplantation^14,17–19^. For example, Lee et al. found that delayed recovery of graft function is an independent risk factor for long-term prognosis after kidney transplantation^17^. Thus, the detection and maintenance of transplanted liver function is the focus of postoperative management after LDLT. However, most of the current studies related to LDLT in children focus on the long-term prognosis, and there is a lack of research on early prognosis, such as recovery of postoperative graft function.

In this study, we included children who underwent LDLT and their relatives who donated liver grafts, analyzed the clinical data of them to explore the influencing factors of early recovery of graft function after LDLT in children.

## Materials and methods

### Patients

We included children who underwent LDLT in our center (a regional medical center for children in China) from January 1, 2018 to June 30, 2023. Inclusion criteria: (1) children with the indication of LDLT, (2) the relatives of the children voluntarily donated part of the liver which met the requirements of LDLT, (3) the therapeutic schedule was approved by the hospital ethics committee and the Red Cross, (4) the written informed consent was obtained. Exclusion criteria: (1) recipients with contraindications of LDLT, (2) failure to complete LDLT due to various reasons (such as death during or within 3 days after operation), (3) recipients or donors with incomplete necessary data, (4) legal guardians of recipients or donors requested to withdraw from this study.

### Variable definition

We obtained the demographic and clinicopathological data of patients included in this study. Preoperative liver function (serological testing, considering AST, ALT and TB) of the donor was categorized as normal or abnormal. Steatosis degree of the liver graft was assessed by two experienced pathologists, divided into four categories: none, microvesicular steatosis, macrovesicular steatosis < 30% and macrovesicular steatosis ≥ 30%. Growth restrictions includes low height (lower than the average height of the same sex and age, more than 2 SDs), low weight (lower than the average weight of the same age and sex, more than 2 SDs) and emaciation (lower than the average weight of the same sex and height, more than 2 SDs), were classified into no and yes. ABO blood type mismatch was defined as matching Rh blood group but not conforming to the principle of ABO blood group transfusion. Graft to recipient weight ratio (GRWR) was calculated from the graft weight and the recipient weight. The ischemic duration of the liver graft was the interval from the occlusion of the donor’s portal vein to the opening of the recipient’s portal vein. According to the average ischemic duration of liver grafts in training cohort, the ischemia duration level of the liver graft was classified into high level and low level. The intraoperative blood loss of the recipient was difficult to quantify, so we used the intraoperative recovery blood volume as the index to evaluate that.

In this study, we used delayed recovery of hepatic function (DRHF) as the outcome variable to evaluate the early recovery of graft function after LDLT in children, which can indicate the impaired ability of the liver to maintain synthesis, excretion or detoxification functions. When the child had elevated PT-INR with hyperbilirubinemia on or after 5 days after LDLT, they were diagnosed as DRHF^20,21^.

### Study design

**Figure 1:**
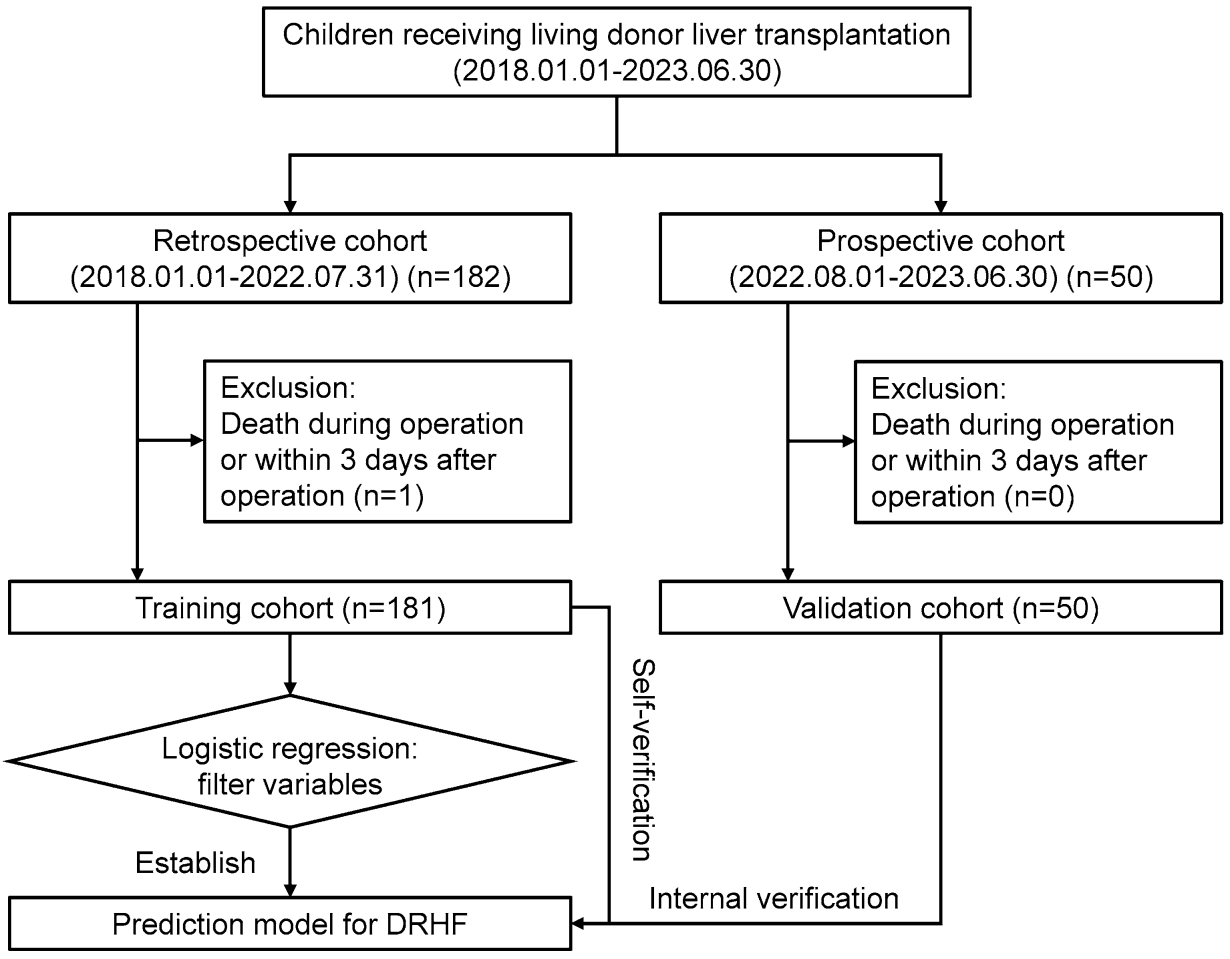
Flowchart of this study.

This is a single-center, ambispective cohort study. According to inclusion criteria and exclusion criteria, we retrospectively included children who underwent LDLT in our center from January 1, 2018 to July 31, 2022 as the training cohort, and prospectively included the group from August 1, 2022 to June 30, 2023 as the validation cohort. We used the training cohort to screen the factors influencing DRHF, which were used to establish a nomogram. We validated the predictive efficacy of the nomogram by itself and internally using the training cohort and validation cohort, respectively, with the area under the curve (AUC) of receiver operating characteristic (ROC) curves , calibration plots, decision curve analysis (DCA) plots and clinical impact curves^22,23^ (Fig. 1). This study performed in accordance with the Declaration of Helsinki, and has been registered in *ClinicalTrials.gov PRS* (Identifiers: NCT06045949, https://www.clinicaltrials.gov/).

### Statistical analysis

Continuous variables with non-normal distribution were represented by “median [P_25_, P_75_]”, *Kruskal–Wallis* test was used to compare the baseline data between groups. Classification variables were showed as “frequency (percentage)”, *Chi-Square* test or *Fisher’s exact* test was used to compare the baseline data between groups. Univariate and multivariate *Logistic* regression analyses were used to analyze the factors affecting DRHF. Variables with *p* < 0.05 in the univariate analysis were used in the multivariate analysis, and variables with *p* < 0.1 and lowest Akaike information criterion (AIC) score in the multivariate analysis were regarded as independent risk factors, which were used to establish the nomogram. Two-sided *p* < 0.05 was statistically significant. All analyses were performed using *tableone*, *rms*, *pROC*, *rmda*, *car*, *ggplot2* and *ggpubr* packages in *R* software (vision 4.0.2).

### Ethical approval

This study has been approved by the Institutional Review Board of Children’s Hospital of Children’s Hospital of Chongqing Medical University , including any relevant details (approval number: 2021-368). All authors confirm that this study was performed in accordance with relevant guidelines and regulations, performed in accordance with the Declaration of Helsinki, and no privacy of donors and recipients was violated in this study. Written informed consent was obtained from the patient’s parents/legal guardian for publication and any clinical data. In addition, no organs/tissues were procured from prisoners in this study.

## Results

### Clinical features

From January 1, 2018 to June 30, 2023, a total of 232 children underwent LDLT in our center, only 1 case was excluded because of intraoperative death, and 231 cases were included in this study (training cohort:validation cohort = 181:50) (Fig. 1). In our center, the donors were the parents of the recipient, and mothers as the donor were more than fathers (60.6% vs. 39.4%). Only a few donors had minimal abnormal liver function (10.4%) and macrovesicular steatosis of the liver (18.2%) before operation. The proportion of male and female recipients was similar (51.1% vs. 48.9%), and a few of them had growth restriction (37.2%). Biliary atresia (BA) was the most common indication of LDLT in our center (87.9%). The ABO blood types of minority recipients and donors did not match (21.2%). With the increase of the operator’s experience, the ischemic duration of the liver graft and the intraoperative blood loss of the recipient decreased significantly (*p* < 0.05). Demographics and clinicopathological characteristics were shown in Table 1.

**Table 1:** Comparison of demographic and clinical characteristics between training and validation cohorts.

	All (n = 231)	Training cohort (n = 181)	Validation cohort (n = 50)	*p* value
Relationship				
Father	91 (39.4%)	72 (39.8%)	19 (38.0%)	0.949
Mother	140 (60.6%)	109 (60.2%)	31 (62.0%)	
Age (donor) (years)				
	29.00 [26.00, 34.00]	30.00 [26.00, 34.00]	28.00 [26.25, 33.75]	0.648
Preoperative liver function (donor)				
Normal	207 (89.6%)	159 (87.8%)	48 (96.0%)	0.158
Abnormal	24 (10.4%)	22 (12.2%)	2 (4.0%)	
Steatosis degree of the liver graft				
None	88 (38.1%)	75 (41.4%)	13 (26.0%)	0.088
Microvesicular steatosis	101 (43.7%)	77 (42.5%)	24 (48.0%)	
Macrovesicular steatosis < 30%	42 (18.2%)	29 (16.0%)	13 (26.0%)	
Macrovesicular steatosis ≥ 30%	0 (0%)	0 (0%)	0 (0%)	
Gender (recipient)				
Male	118 (51.1%)	98 (54.1%)	20 (40.0%)	0.107
Female	113 (48.9%)	83 (45.9%)	30 (60.0%)	
Age (recipient) (months)				
	5.67 [4.97, 6.95]	5.57 [4.80, 7.10]	5.72 [5.31, 6.52]	0.263
Growth restriction (recipient)				
No	145 (62.8%)	114 (63.0%)	31 (62.0%)	1
Yes	86 (37.2%)	67 (37.0%)	19 (38.0%)	
ABO blood type matching				
Matching	182 (78.8%)	146 (80.7%)	36 (72.0%)	0.258
Mismatching	49 (21.2%)	35 (19.3%)	14 (28.0%)	
Indication				
BA	203 (87.9%)	159 (87.8%)	44 (88.0%)	0.403
CTPV	7 (3.0%)	7 (3.9%)	0 (0.0%)	
Congenital metabolic liver disease	18 (7.8%)	13 (7.2%)	5 (10.0%)	
Others	3 (1.3%)	2 (1.1%)	1 (2.0%)	
PELD score				
	14.50 [8.88, 19.50]	14.26 [8.60, 19.51]	15.58 [10.37, 19.42]	0.699
Child Pugh Score				
	8.00 [7.00, 9.00]	8.00 [7.00, 9.00]	8.00 [7.00, 9.00]	0.851
GRWR (%)				
	3.43 [2.79, 4.22]	3.40 [2.80, 4.24]	3.49 [2.76, 3.95]	0.727
Ischemia duration level of the liver graft				
Low level	155 (67.1%)	107 (59.1%)	48 (96.0%)	< 0.001
High level	76 (32.9%)	74 (40.9%)	2 (4.0%)	
Operation duration (recipient) (min)				
	455.00 [410.00, 500.00]	455.00 [410.00, 510.00]	453.50 [412.50, 483.25]	0.418
Recovery blood volume (recipient) (mL)				
	320.00 [200.00, 510.50]	355.00 [230.00, 580.00]	220.00 [168.50, 300.00]	< 0.001
DRHF				
No	163 (70.6%)	125 (69.1%)	38 (76.0%)	0.437
Yes	68 (29.4%)	56 (30.9%)	12 (24.0%)	

*BA* biliary atresia, *CPTV* cavernous transformation of portal vein, *DRHF* delayed recovery of hepatic function, *GRWR* Graft to recipient weight ratio, *PELD* Pediatric end-stage liver disease score.

### Analysis of influence on early recovery of graft function after operation

**Figure 2:**
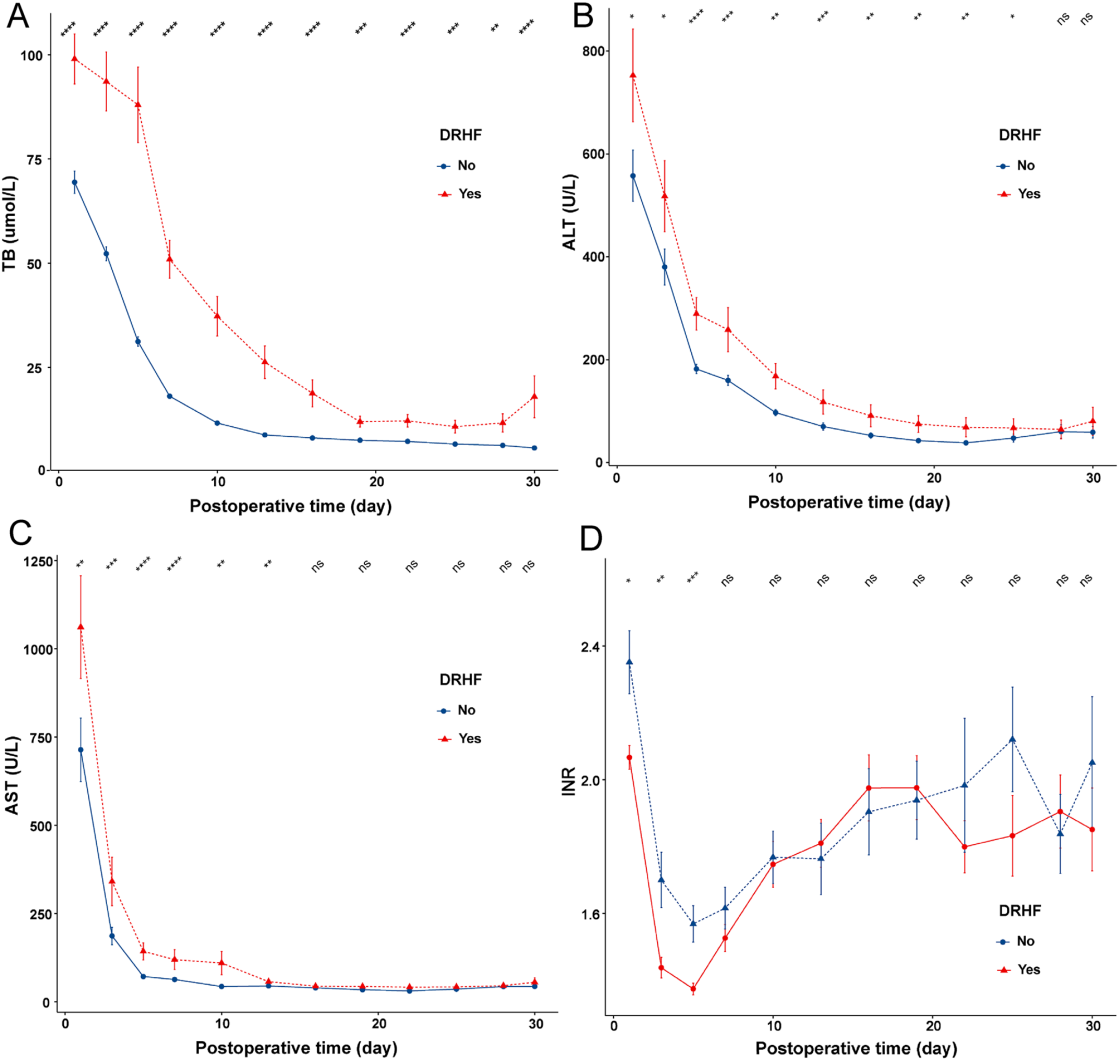
Changes of early graft function after LDLT in children. (**A**) postoperative TB changes in different DRHF groups. (**B**) postoperative ALT changes in different DRHF groups; (**C**), postoperative AST changes in different DRHF groups. (**D**), postoperative INR changes in different DRHF groups. ns *p* > 0.05, * 0.05 > *p* > 0.01, ** 0.01 > *p* > 0.001, *** 0.001 > *p* > 0.0001, *** *p* < 0.0001.

In this study, a few recipients had DRHF after operation (29.4%). The incidence of DRHF in the prospective cohort was lower than in the retrospective cohort (24.0% vs. 30.9%), possibly due to advances in surgical techniques and management experience (Table 1). We drew line charts of early postoperative graft function changes in all recipients included in this study. We found that early postoperative graft function recovery in the DRHF group was significantly worse than that in the no DRHF group (Fig. 2). The correlation analysis of DRHF and early postoperative complications showed that DRHF group had higher incidence of early postoperative complications, especially bile leakage and stenosis of anastomotic vessels (*p* < 0.05, Table 2). In addition, we found that the postoperative hospital stay in the DRHF group was significantly less than that in the no DRHF group (*p* < 0.05, Supplementary material, Table S1 and Fig. S1). Thus, the above results suggested that DRHF could be used to evaluate the speed of early recovery of recipient abnormal graft function after operation.

**Table 2:** Relationship between early postoperative complications and DRHF.

	All (n = 231)	no DRHF (n = 163)	DRHF (n = 68)	*p* value
Wound infection				
No	224 (97.0%)	159 (97.5%)	65 (95.6%)	0.423
Yes	7 (3.0%)	4 (2.5%)	3 (4.4%)	
Urine infection				
No	225 (97.4%)	159 (97.5%)	66 (97.1%)	1
Yes	6 (2.6%)	4 (2.5%)	2 (2.9%)	
Chest infection				
No	148 (64.1%)	110 (67.5%)	38 (55.9%)	0.1
Yes	83 (35.9%)	53 (32.5%)	30 (44.1%)	
Abdominal infection				
No	206 (89.2%)	150 (92.0%)	56 (82.4%)	0.038
Yes	25 (10.8%)	13 (8.0%)	12 (17.6%)	
Bacteraemia				
No	223 (96.5%)	157 (96.3%)	66 (97.1%)	1
Yes	8 (3.5%)	6 (3.7%)	2 (2.9%)	
Fungal infection				
No	216 (93.5%)	153 (93.9%)	63 (92.6%)	0.772
Yes	15 (6.5%)	10 (6.1%)	5 (7.4%)	
Portal vein thrombosis				
No	227 (98.3%)	160 (98.2%)	67 (98.5%)	1
Yes	4 (1.7%)	3 (1.8%)	1 (1.5%)	
Hepatic artery thrombosis				
No	230 (99.6%)	163 (100.0%)	67 (98.5%)	0.294
Yes	1 (0.4%)	0 (0.0%)	1 (1.5%)	
Bile leakage				
No	226 (97.8%)	162 (99.4%)	64 (94.1%)	0.027
Yes	5 (2.2%)	1 (0.6%)	4 (5.9%)	
Biliary stenosis				
No	227 (98.3%)	162 (99.4%)	65 (95.6%)	0.078
Yes	4 (1.7%)	1 (0.6%)	3 (4.4%)	
Hemadostenosis				
No	209 (90.5%)	153 (93.9%)	56 (82.4%)	0.012
Yes	22 (9.5%)	10 (6.1%)	12 (17.6%)	
Hematorrhea				
No	225 (97.4%)	161 (98.8%)	64 (94.1%)	0.064
Yes	6 (2.6%)	2 (1.2%)	4 (5.9%)	
Acute rejection				
No	226 (97.8%)	160 (98.2%)	66 (97.1%)	0.633
Yes	5 (2.2%)	3 (1.8%)	2 (2.9%)	
Acute kidney injury				
No	229 (99.1%)	163 (100.0%)	66 (97.1%)	0.086
Yes	2 (0.9%)	0 (0.0%)	2 (2.9%)	
Hemodiafiltration				
No	231 (100.0%)	163 (100.0%)	68 (100.0%)	/
Yes	0 (0.0%)	0 (0.0%)	0 (0.0%)	
Retransplanted				
No	229 (99.1%)	162 (99.4%)	67 (98.5%)	0.503
Yes	2 (0.9%)	1 (0.6%)	1 (1.5%)	
Death				
No	228 (98.7%)	162 (99.4%)	66 (97.1%)	0.208
Yes	3 (1.3%)	1 (0.6%)	2 (2.9%)	

The results of univariate *Logistic* regression analysis showed that preoperative liver function of donors [OR, 3.136, 95% CI (1.265–7.940)], age of recipients [OR, 1.018, 95% CI (1.004–1.034)], *Ln* (ALB of recipients before operation [OR, 0.050, 95% CI (0.008–0.289)], *Ln* (ALT of recipients before operation) [OR, 0.675, 95% CI (0.469–0.962)], *Ln* (Cr of recipients before operation) [OR, 0.734, 95% CI (0.508–1.056)], ischemia duration level of the liver graft [OR, 3.284, 95% CI (1.719–6.396)], operation duration of recipients [OR, 1.005, 95% CI (1.001–1.008)], recovery blood volume of recipients [OR, 1.002, 95% CI (1.001–1.004)], *Ln* (TB of recipients on the 1st day after operation) [OR, 3.183, 95% CI (1.675–6.543)], *Ln* (AST of recipients on the 1st day after operation) [OR, 1.964, 95% CI (1.219–3.259)], *Ln* (TB of recipients on the 3rd day after operation) [OR, 9.592, 95% CI (4.269–24.459)], *Ln* (AST of recipients on the 3rd day after operation) [OR, 1.880, 95% CI (1.238–2.951)], *Ln* (Cr) of recipients on the 3rd day after operation) [OR, 2.627, 95% CI (1.132–6.269)] were closely related with DRHF of children after LDLT (*p* < 0.05, Table 3). And then, multivariate *Logistic* regression analysis showed that preoperative liver function of donors [OR, 3.618, 95% CI (1.111–12.189)], ischemia duration level of the liver graft [OR, 2.664, 95% CI (1.083–6.781)] and *Ln* (TB of recipients on the 3rd day after operation) [OR, 7.433, 95% CI (2.366–26.996)] significantly affected DRHF of children after LDLT (*p* < 0.05, Table 3).

**Table 3:** Univariate and multivariate *Logistic* regression analyses to identify risk factors for DRHF.

	Univariate analysis		Multivariate analysis	
	OR (95% CI)	*p* value	OR (95% CI)	*p* value
Relationship				
Father	1.000 (Reference)	–	/	/
Mother	0.925 (0.488–1.770)	0.812	/	/
Age (donor) (years)				
	1.045 (0.992–1.102)	0.098	/	/
Preoperative liver function (donor)				
Normal	1.000 (Reference)	–	1.000 (Reference)	–
Abnormal	3.136 (1.265–7.940)	0.014	3.618 (1.111–12.189)	0.034
Steatosis degree of the liver graft				
None	1.000 (Reference)	–	/	/
Microvesicular steatosis	0.750 (0.372–1.500)	0.417	/	/
Macrovesicular steatosis < 30%	1.053 (0.415–2.569)	0.911	/	/
Macrovesicular steatosis ≥ 30%	/	/	/	/
Gender (recipient)				
Male	1.000 (Reference)	–	/	/
Female	1.147 (0.609–2.161)	0.670	/	/
Age (recipient) (months)				
	1.018 (1.004–1.034)	0.016	1.008 (0.979–1.040)	0.618
Growth restriction (recipient)				
No	1.000 (Reference)	–	/	/
Yes	1.150 (0.597–2.194)	0.672	/	/
ABO blood group matching				
Matching	1.000 (Reference)	–	/	/
Mismatching	0.495 (0.188–1.158)	0.124	/	/
*Ln* (TB) (recipient, before operation) (μmol/L)				
	0.907 (0.694–1.200)	0.478	/	/
*Ln* (ALB) (recipient, before operation) (g/L)				
	0.050 (0.008–0.289)	0.001	0.167 (0.004–6.258)	0.336
*Ln* (ALT) (recipient, before operation) (U/L)				
	0.675 (0.469–0.962)	0.031	0.945 (0.519–1.723)	0.853
*Ln*(AST) (recipient, before operation) (U/L)				
	0.734 (0.508–1.056)	0.095	/	/
*Ln* (Cr) (recipient, before operation) (μmol/L)				
	3.032 (1.068–8.775)	0.037	6.243 (0.940–44.736)	0.060
PELD score				
	1.028 (0.998–1.061)	0.074	/	/
Child Pugh Score				
	1.351 (1.133–1.626)	0.001	1.037 (0.719–1.496)	0.844
GRWR (%)				
	0.895 (0.669–1.190)	0.447	/	/
Ischemia duration level of the liver graft				
Low level	1.000 (Reference)	–	1.000 (Reference)	–
High level	3.284 (1.719–6.396)	3.723E–04	2.664 (1.083–6.781)	0.035
Operation duration (recipient) (min)				
	1.005 (1.001–1.008)	0.008	0.998 (0.993–1.003)	0.436
Recovery blood volume (recipient) (mL)				
	1.002 (1.001–1.004)	2.543E−04	1.001 (1.000–1.003)	0.321
*Ln* (TB) (recipient, the 1st day after operation) (μmol/L)				
	3.183 (1.675–6.543)	8.418E−04	1.424 (0.540–4.106)	0.493
*Ln* (ALB) (recipient, the 1st day after operation) (g/L)				
	0.606 (0.135–2.813)	0.513	/	/
*Ln* (ALT) (recipient, the 1st day after operation) (U/L)				
	1.529 (0.942–2.518)	0.088	/	/
*Ln* (AST) (recipient, the 1st day after operation) (U/L)				
	1.964 (1.219–3.259)	0.007	1.607 (0.588–4.487)	0.357
*Ln* (Cr) (recipient, the 1st day after operation) (μmol/L)				
	2.336 (0.987–5.585)	0.053	/	/
*Ln* (TB) (recipient, the 3rd day after operation) (μmol/L)				
	9.592 (4.269–24.459)	3.330E–07	7.433 (2.366–26.996)	0.001
*Ln* (ALB) (recipient, the 3rd day after operation) (g/L)				
	1.174 (0.126–11.578)	0.888	/	/
*Ln* (ALT) (recipient, the 3rd day after operation) (U/L)				
	1.394 (0.895–2.182)	0.140	/	/
*Ln* (AST) (recipient, the 3rd day after operation) (U/L)				
	1.880 (1.238–2.951)	0.004	1.219 (0.515–2.889)	0.648
*Ln* (Cr) (recipient, the 3rd day after operation) (μmol/L)				
	2.627 (1.132–6.269)	0.026	1.464 (0.397–5.417)	0.565

*ALB* serum albumin, *ALT* alanine aminotransferase, *AST* aspartate aminotransferase, *Cr* creatinine, *DRHF* delayed recovery of hepatic function, *GRWR* graft to recipient weight ratio, *PELD* pediatric end-stage liver disease score, *TB* total bilirubin.

### Prediction model construction and verification

We found that *p* value of *Ln* (Cr of recipients before operation) was close to 0.05 in multivariate analysis, so we calculated the AIC score. The results suggested that the score of AIC was lower after adding *Ln* (Cr of recipients before operation) (170.07 vs. 179.27), which indicated better discriminatory ability^24^. Therefore, preoperative liver function of donors, ischemia duration level of the liver graft, *Ln* (Cr of recipients before operation) and *Ln* (TB of recipients on the 3rd day after operation) were used to build a model to predict the probability of DRHF in children after LDLT, as shown below:

$$\begin{aligned} {\text{Logit}}\left( {\text{P}} \right) &  = - {17}.{57}0 \, + { 1}.{218} \times {\text{preoperative liver function of donors }} + {1}.{448} \\ & \quad  \times {\text{ischemia duration level of the liver graft }} + { 2}.{161 } \times Ln({\text{Cr ofrecipients before operation}}) \\ & \quad  + { 2}.{388} \times Ln\left( {{\text{TB of recipients on the 3rd}} {\text{day after operation}}} \right) \end{aligned}$$

Note. (1) Preoperative liver function of donors: normal is 0, abnormal is 1. (2) Ischemia duration level of the liver graft: low level (< 106.97 min) is 0, high level (> = 106.97) is 1.

**Figure 3:**
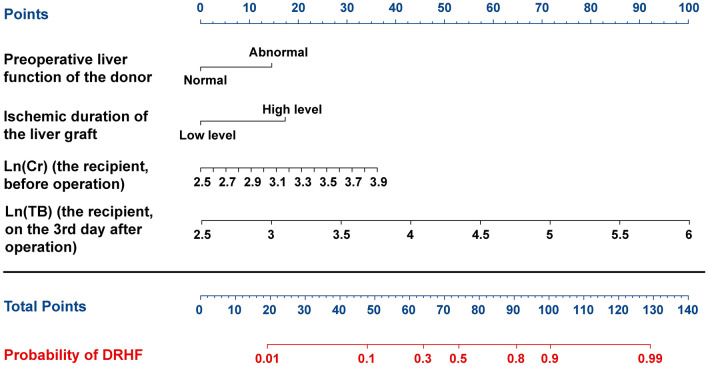
Nomogram for predicting DRHF in children after LDLT. The units of Cr is μmol/L; the units of TB is μmol/L.

**Figure 4:**
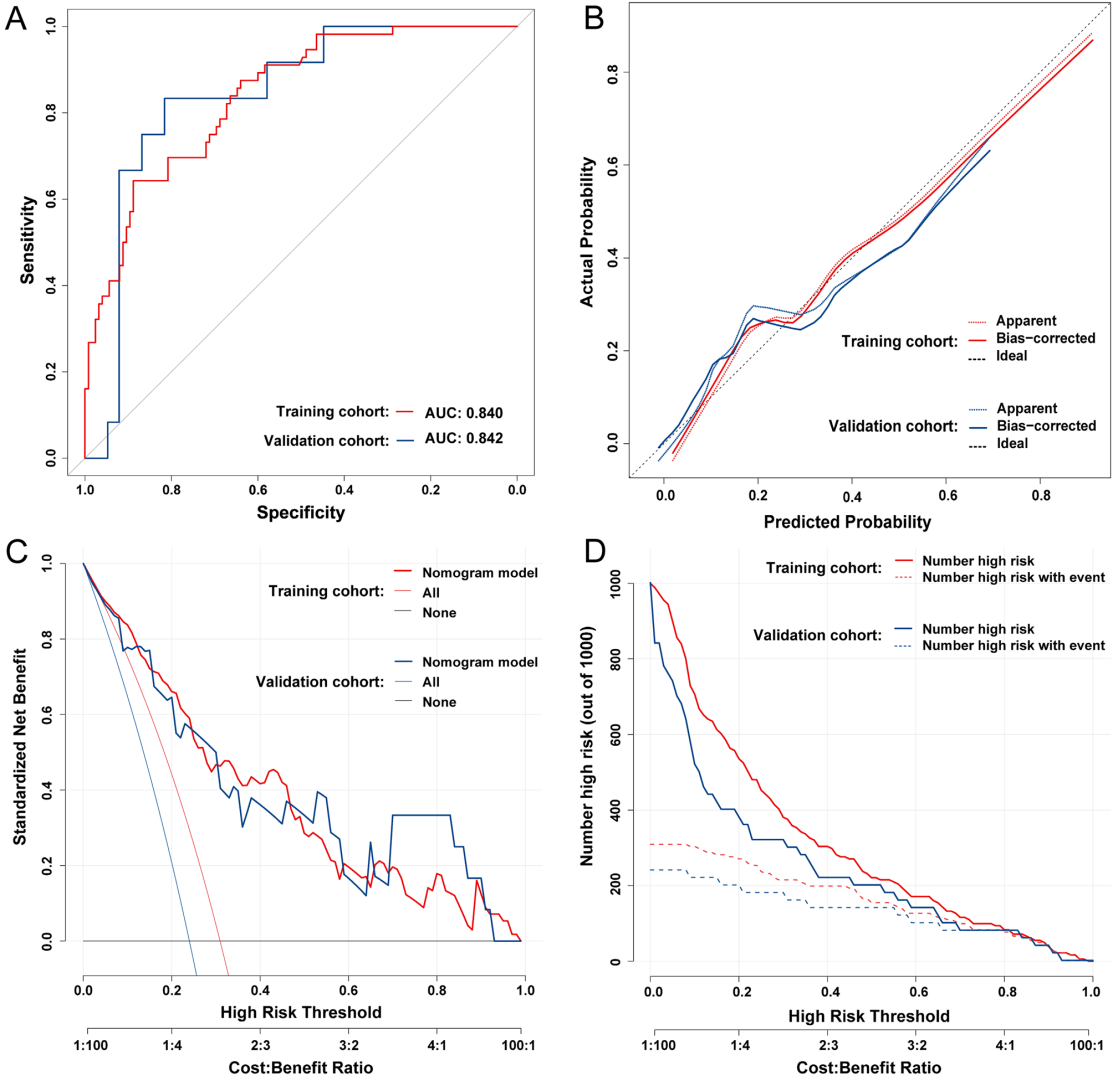
Self-verification and internal verification of nomogram for predicting DRHF in children after LDLT. (**A**) ROC curves in training and validation cohorts. (**B**) Calibration curves in training and validation cohorts. (**C**) DCA plots in training and validation cohorts. (**D**) clinical impact curves in training and validation cohorts.

To make it more intuitive to calculate the probability of DRHF occurrence, we constructed a nomogram (Fig. 3). We verified our prediction model by itself and internally using the training cohort and validation cohort, respectively. The AUCs of DRHF prediction results in training and validation cohorts were 0.840 and 0.842, respectively, which showed that our prediction model had better discriminatory ability (Fig. 4a). Calibration curves showed that the predicted results were uniformly and closely distributed around the ideal line in training and validation cohorts, suggesting that the predicted results were in good agreement with the actual outcomes (Fig. 4b). In addition, DCA plots and clinical impact curves showed that patients using our model to predict the probability of DRHF could obtain a good net benefit, which showed that our prediction model had good clinical applicability (Fig. 4c,d).

## Discussion

In this study, based on a single-center ambispective cohort of LDLT in children, we found that DRHF could be a good indicator of early postoperative graft function recovery. Preoperative liver function of donors, ischemia duration level of the liver graft, *Ln* (Cr of recipients before operation) and *Ln* (TB of recipients on the 3rd day after operation) were closely related to the early recovery of postoperative graft function. Using the above factors, we established a nomogram with good performance to predict DRHF of children after LDLT.

LT as the gold-standard treatment for end-stage liver disease in children can be used to treat cholestatic liver disease, metabolic liver disease, acute liver failure, neoplastic disease, vascular disease, re-transplantation and others^9,10^. Pediatric LDLT remains infrequently performed in Occident, deceased donors making up most LT performed^25^. However, LDLT in children as one of the most mainstream treatment options performs frequently in the eastern countries. In the South Korea, LDLT accounted for 76.5% of all LTs from 1988 to 2013^26^. In this center, LDLT accounted for more than 80% of pediatric LTs in the past five years, and the leading LT indication was BA (87.9%).

The monitoring and maintenance of postoperative graft function is the key to patient management after LT, which is not only related to the survival rate of liver graft and the decision of the immunosuppressive regimen, but also an important evaluation index of clinical researches^27–29^. Many studies have shown that early graft function (such as ALT, AST, INR, and TB) after LT (including adult LT, LDLT, pediatric LT) was closely related to long-term survival rates of patients, graft survival rates, disease recurrence and other long-term prognosis, that have confirmed the important research value of early recovery of graft function after LT^30–33^. However, there is a lack of researches on the early recovery of graft function after LDLT in children, so there is no generally accepted and applied definition or grading system to evaluate it. Studies have found that DRHF can be used to evaluate liver failure after hepatectomy, and be used to evaluate the early recovery of graft function after LDLT^20,21^. In this study, we found that DRHF can evaluate the recovery status of recipient abnormal graft function after LDLT in children, too. At present, only one study reported that CT-based liver volumetry can evaluate the recovery of native liver function recovery after auxiliary partial LT in patients with acute liver failure^34^. In this study, we found that preoperative donor liver insufficiency, recipient preoperative higher creatinine, longer ischemia time of the liver graft and early postoperative higher bilirubin were risk factors for DRHF, which can be used to evaluate the early recovery of graft function after LDLT in children. Coincidentally, all above factors were also closely related to the long-term survival rate and graft survival rate after adult LT, pediatric LT^32,35–37^. Therefore, we believe that the abnormality of the above indexes should be paid attention to in the management of children after LDLT.

Personalized management is an important development direction of modern medical model, which not only helps to reduce excessive medical intervention, but also helps to improve doctor-patient communication^22^. As a visual, personalized and convenient tool, the nomogram has been increasingly used in medical research, such as predicting the prognosis of malignancies, treatment efficacy and other medical activities^38–40^. However, there are only a few application researches of predictive models in pediatric LT, such as predicting the length of length of stay in intensive care unit, the incidence of late-onset acute cellular rejection, the incidence of ischemic-type biliary lesions and postoperative survival rate^41–45^. In this study, we constructed the first predictive model with several simple indicators to evaluate the early recovery of graft function in children after LDLT. Self-verification and prospective internal verification showed that this prediction model had good prediction accuracy and clinical application value. The importance of detecting early postoperative graft function recovery has been explained. This predictive tool may be used to predict the risk of graft dysfunction, so as to focus earlier attentions and take timely intervention measures to rescue grafts as soon as possible, such as puncture biopsy, changing immunosuppressive regimens.

This study has several limitations still. First, this study is a single-center cohort study, there are some limitations, such as small sample size, incomplete inclusion factors and selection bias. In addition, we only used single-center data to build the prediction model and performed self-verification and internal verification, lack of external verification using multi-center data, which could affect the extensibility of this prediction model. Because we’ve only been carrying out a lot of LDLT in recent years, the follow-up time in this study was short. Thus, we can’t explore the relationship between the recovery of postoperative graft function and the long-term prognosis of patients. In the future, we will further carry out a multicenter, large sample study on the basis of this study, verify and expand the application value of this study, and further explore the correlation between short-term prognosis and long-term prognosis to reveal the importance of short-term prognosis.

## Conclusion

In summary, DRHF is a good indicator of postoperative graft function recovery of children after LDLT. We have demonstrated risk factors for early delayed recovery of liver function after LDLT in children, including preoperative donor liver insufficiency, recipient preoperative higher creatinine, longer ischemia duration of the liver graft and early postoperative higher bilirubin. Based on our finding, we established the first prediction model with good performance which could help clinicals early evaluate the recovery of liver function after pediatric LDLT earlier, so that patients can be treated or managed more pointedly and appropriately.

### Supplementary Information

Supplementary Information.

## Data Availability

The data analyzed during this current study are available from corresponding authors upon reasonable request.

## Abbreviations

ALB: Serum albumin
AIC: Akaike information criterion
ALT: Alanine aminotransferase
AST: Aspartate aminotransferase
AUC: Area under the curve
BA: Biliary atresia
CPTV: Cavernous transformation of portal vein
Cr: Creatinine
DCA: Decision curve analysis
DRHF: Delayed recovery of hepatic function
GRWR: Graft to recipient weight ratio
LDLT: Living donor liver transplantation
LT: Liver transplantation
PELD: Pediatric end-stage liver disease score
PT-INR: Prothrombin time international normalized ratio
ROC: Receiver operating characteristic
SD: Standard deviation
TB: Total bilirubin
