# Supplementary material for: Prediction of early recovery of graft function after living donor liver transplantation in children
Source: Sci Rep. 2024 Apr 24;14:9472. doi: 10.1038/s41598-024-60211-6 (PMC11043388; doi:10.1038/s41598-024-60211-6)
Supplement: Supplementary file 1 — Supplementary Information. [file 41598_2024_60211_MOESM1_ESM.docx]

# Supplementary materials

**Table S1. The difference of postoperative hospital stay in recipients with and without DRHF**

|  | **All (n=231)** | **no DRHF (n=163)** | **DRHF (n=68)** | ***p* value** |
| --- | --- | --- | --- | --- |
| **Postoperative hospital stay (day)** | 26 [22, 34] | 25 [21, 33] | 28 [23, 36] | 0.039 |

**Figure S1. The difference of postoperative hospital stay in recipients with and without DRHF**


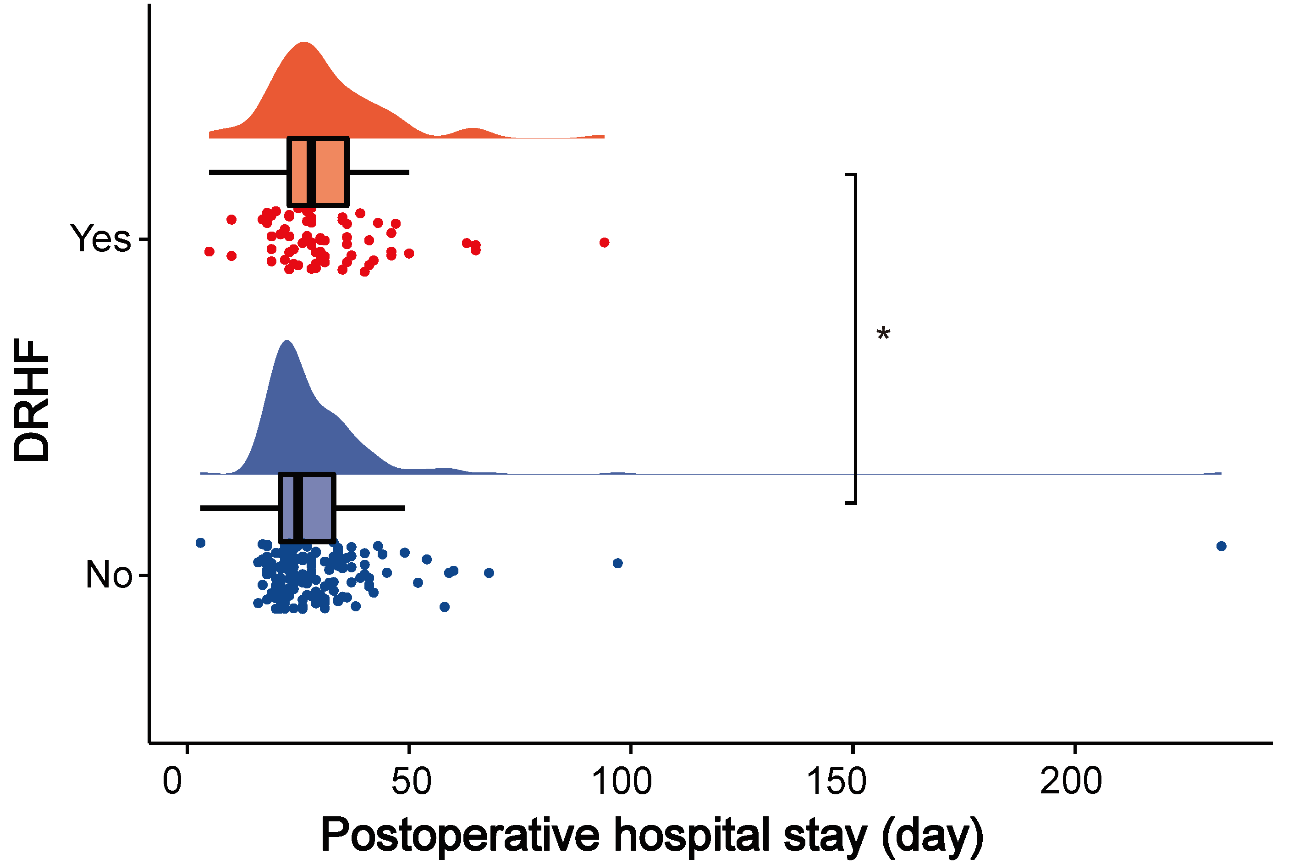


**Note.** ns *p* >0.05, * 0.05 > *p* >0.01, ** 0.01 > *p* >0.001, *** 0.001 > *p* >0.0001, *** *p* < 0.0001.
